# Easy and Effective Method for Extracting and Purifying *Wolbachia* Genomic DNA

**DOI:** 10.3390/ijms232315315

**Published:** 2022-12-05

**Authors:** Olga V. Andreenkova, Olga D. Shishkina, Alexandra I. Klimenko, Aleksandra E. Korenskaia, Margarita A. Bobrovskikh, Natalja V. Shatskaya, Gennady V. Vasiliev, Nataly E. Gruntenko

**Affiliations:** 1 Institute of Cytology and Genetics SB RAS, Novosibirsk 630090, Russia; 2 Kurchatov Genomics Center, Institute of Cytology and Genetics, Siberian Branch of the Russian Academy of Science, Lavrentiev Avenue 10, Novosibirsk 630090, Russia; 3 Department of Natural Sciences, Novosibirsk National Research State University, Pirogova St. 1, Novosibirsk 630090, Russia

**Keywords:** *Wolbachia*, genome DNA extraction, *Drosophila melanogaster*

## Abstract

A number of methods for extracting the DNA of maternally inherited obligate intracellular bacteria *Wolbachia* from an insect host and its subsequent purification have been described in previous scholarship. As *Wolbachia* is present in the hosts’ organisms in rather low quantities, these techniques used to be quite labor-intensive. For this paper, we analyzed them in detail, searched for a possibility to simplify and accelerate the protocol, and proposed an easy and effective method for isolating *Wolbachia* DNA from *Drosophila melanogaster* with a purity sufficient for genomic sequencing. Our method involves the centrifugation of homogenized flies or just their ovaries, as the most *Wolbachia*-enriched tissue, followed by the filtration of homogenate and extraction of DNA using a modified version of the Livak buffer protocol. The proportion of *Wolbachia* DNA in the total DNA was quantified based on the results of sequencing with the use of the Illumina MiSeq platform and a pipeline of bioinformatic analysis. For the two analyzed *D. melanogaster* lines infected with two different *Wolbachia* strains, the proportion was at least 68 and 94%, respectively.

## 1. Introduction

*Wolbachia* are intracellular gram-negative bacteria, which are found to infect many invertebrate species including insects, ticks, spiders, terrestrial crustaceans and nematodes. *Wolbachia* transmit transovarially from females to their offspring and are extremely widespread: they were found to infect 40% of the analyzed invertebrate species [1]. *Wolbachia* are able to manipulate the host’s reproduction, thereby increasing their transmission rate in an infected population. The manipulation strategies include feminization, male loss, parthenogenesis and cytoplasmic incompatibility [2,3,4]. Some strains that successfully invade the hosts’ populations do not exhibit a capacity to manipulate the host’s reproduction system. It is assumed that such strains spread in a population due to their ability to grant an advantage to the host’s fitness. The prevalence of *Wolbachia* not only in reproductive but also in somatic tissues such as the fat body and brain is usually linked with various positive effects that these bacteria produce in somatic processes of the host organism. For instance, it was shown that *Wolbachia* reduce the susceptibility of *Drosophila* to viral infections [5,6,7]. Moreover, there is evidence for *Wolbachia’s* impact on the size of *Drosophila melanogaster* host insects [8] and their life span [9,10,11].

Since *Wolbachia* is an obligate intracellular symbiont, it is impossible to cultivate it outside host cells. This impedes the extraction from *Wolbachia* of DNA that would be pure enough for genomic studies. A number of techniques have been developed allowing the extraction of *Wolbachia* DNA suitable for sequencing from insects such as *Drosophila* and mosquitoes. However, these techniques are usually quite laborious and in most cases demand a large amount of source material.

The pioneering work on *Wolbachia* DNA extraction for genomic studies was conducted by Sun et al. [12], who developed a DNA extraction method for *Wolbachia* free from the host’s mitochondrial DNA while aiming to determine the genome size of six different *Wolbachia* strains from insect and nematode hosts. This method uses differential centrifugation of a homogenate from thousands of entire host organisms to extract *Wolbachia* cells and monitor their genome by pulsed-field gel electrophoresis (PFGE).

Mavingui et al. [13] suggested a way to extract large quantities of high-quality *Wolbachia* DNA for genomic analysis from *D. simulans* eggs, supplementing the differential centrifugation technique of DNA excision after PFGE with the procedure of whole-genome amplification by multiple-displacement amplification (MDA). The authors obtained sufficient amounts (8 to 10 µg) of DNA generated by MDA of suitable quality for genomic studies.

Subsequently, three methods of *Wolbachia* DNA extraction without the use of PFGE were suggested. For better purification of *Wolbachia* from nuclei and mitochondria of the host organism, differential centrifugation was supplemented by filtration through a filter with a pore size which allowed *Wolbachia* to pass through, but not intact nuclei and mitochondria of the host. Thus, Klasson et al. [14] used filters with 5 µm pore size for *Wolbachia* excretion from 50 preblastoderm embryos of *Culex pipiens* mosquitoes. The filtrate was centrifuged at 5000× *g* (4 °C) for 15 min to pellet the *Wolbachia*. As the mean total amount of DNA resulting from an individual extraction was 408.8  ±  102.6 ng, 50 extractions were pooled to produce a sufficient amount of DNA for genome sequencing.

Iturbe-Ormaetxe et al. [15] developed another method of extracting *Wolbachia* DNA from imago of *D. simulans, D. melanogaster* and *Aedes aegypti* mosquitoes highly purified from host DNA. Their innovation comprised three successive filtrations of a homogenate containing *Wolbachia* cells through filters with a pore diameter of 5 µm or less. To remove DNA contamination, the samples were treated with DNAseI. It is important to note that the obtained concentration of *Wolbachia* DNA was 100–200 μg when measured by NanoDrop spectrophotometer and 10 times lower when using a fluorimeter [15]. The sequences produced by this method were highly enriched in *Wolbachia*, typically producing data sets in which 90–97% of the raw reads were *Wolbachia* sequence. However, it is necessary to use a large number of insects, which might be considered a disadvantage of this method.

The latest published method for extracting *Wolbachia* from insect hosts was developed by Ellegaard et al. [16]. *Wolbachia* cells were purified from embryos of *D. simulans* using a combination of steps of differential centrifugation, filtration and multiple-displacement amplification (MDA) performed directly on the bacterial pellet. The homogenate was prepared from 15–30 dechorionized embryos in a phosphate buffer. Differential centrifugation was performed at 4 °C as in Klasson et al. [14], with minor modifications; successive filtering through filters with different pore sizes was performed as in Iturbe-Ormaetxe et al. [15]. It is worth noting that although this method makes it possible to obtain a high-quality *Wolbachia* genomic DNA (97–99% of the raw reads were *Wolbachia*) from a small amount of host material, it also includes MDA and is quite complicated technically.

Based on these methods, our aim here was to find the fastest and easiest way to isolate *Wolbachia* DNA of sufficient quality and quantity for sequencing from a relatively small amount of *D. melanogaster* imagoes.

## 2. Results and Discussion

Three pairs of samples were used for establishing the method we present here. For the first two pairs of samples, we isolated *Wolbachia* DNA from 50 ovaries of female fruit flies with or without the use of a filter. For the third pair of samples we took the whole insect as a source of *Wolbachia* DNA, instead of only the ovaries, used a filter and increased the number of females up to 150 per sample. An advantage of this approach is the possibility to quickly extract *Wolbachia* DNA from a small number of flies. This significantly simplifies the protocol compared to the methods of Sun et al. [12], who used about 1000 flies to isolate Wolbachia, or Iturbe-Ormaetxe et al. [15], who took even more specimens for one sample—up to 5000.

At the first stage of the approbation of our method we extracted the DNA from the *D. melanogaster* ovaries—the tissue enriched in *Wolbachia* cells. We picked up the idea of using Wolbachia-enriched material as a source of bacteria from the studies by Mavingui et al. [13] and Ellegaard et al. [16], who used *D. simulans* embryos for this purpose. However, the usage of embryos demands dechorionization, which seems to be more labor-intensive than the dissection of females in order to obtain the ovaries. This approach can also grant an advantage to the investigator if he or she needs to extract *Wolbachia* from flies of a certain genotype, which is supported by using a balancer chromosome. One can select the females of a certain phenotype when isolating the ovaries but cannot do this with eggs.

**Figure 1:**
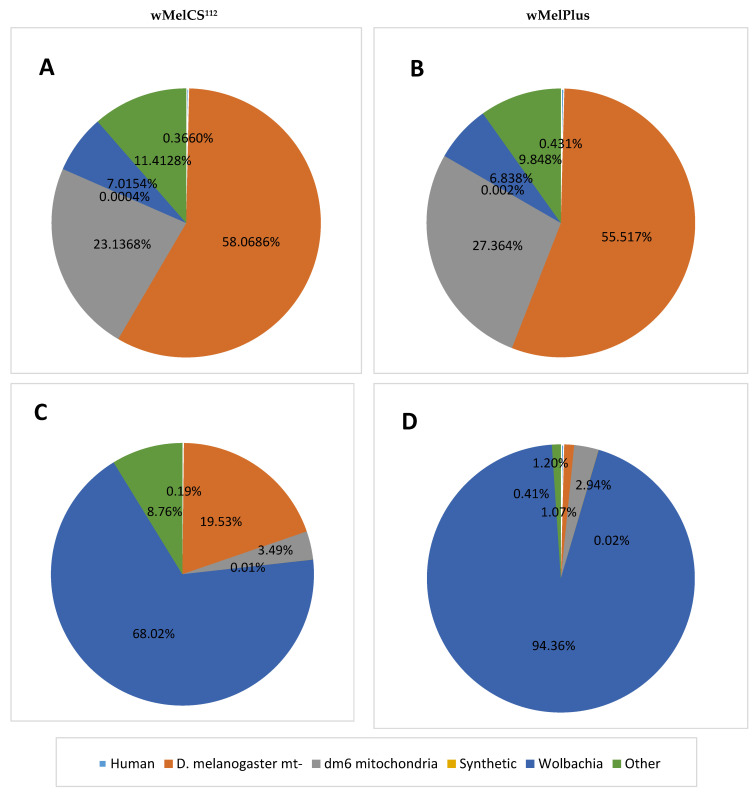
The fractions of reads, belonging to different sources in the four sequenced samples containing *Wolbachia* strains wMelCS^112^ (**A**,**C**) and wMelPlus (**B**,**D**) extracted from *D. melanogaster* ovaries without use of a filter (**A**,**B**) and from whole females with the use of a filter (**C**,**D**). Human—hg38 human genome assembly; *D. melanogaster* mt—*Drosophila* dm6 genome assembly, excluding mitochondria; dm6 mitochondria—mitochondrial genome taken from the *Drosophila* dm6 genome assembly; synthetic—synthetic sequences included in the UniVec database; *Wolbachia*—*Wolbachia* wMelCS_b genome assembly; other—the other reads of unknown origin.

Then we used differential centrifugation for the sedimentation of the host’s nuclei to sufficiently purify *Wolbachia* from the host’s DNA as in Klasson et al. [14] and Ellegaard et al. [16] and assessed the quality of extracted DNA via sequencing (Figure S1A,B). For the purpose of filtering the sequenced data, potentially belonging *to Wolbachia pipientis* from other biological sources, contamination control was consecutively conducted for the human sequences (hg38), *Drosophila melanogaster* (dm6—separately nuclear and mitochondrial genomes) and synthetic sequences included in the UniVec database (for details see Section 3.3). However, the sequencing of extracted samples showed a substantial contamination with the nuclear and mitochondrial DNA of the host. The largest portion of reads from the whole sequenced data belonged to the genome of *D. melanogaster*, and only 18.43% of reads might contain the sequences of wMeCS^112^ (Figure S1A) and 16.69% of reads—the sequences of wMelPlus (Figure S1B) strains of *Wolbachia*. Applying stricter estimates shows that only 7.02% of reads map onto the *Wolbachia* reference genome for wMelCS^112^ (Figure 1A) and 6.84% of reads for wMelPlus (Figure 1B) strains of *Wolbachia*.

Next, we added the stage of filtering the homogenate containing *Wolbachia* through a syringe filter with a pore diameter of 5 μm to the protocol, as in Klasson et al. [14]. The stages of filtering through filters with smaller pore sizes followed Iturbe-Ormaetxe et al. [15] and Ellegaard et al. [16]. DNase and RNase treatments used by Iturbe-Ormaetxe et al. [15] were also omitted in our practice in order to minimize the loss of Wolbachia DNA as well as speed up and simplify the process of its purification. However, the amount of DNA obtained from 50 pairs of ovaries turned out to be too small for sequencing (<10 ng, Qubit Fluorometric Quantification, Thermo Fisher Scientific, Waltham, MA, USA).

Since the extraction of ovaries in large amounts is an arduous and time-consuming process, we adapted the protocol for the extraction of *Wolbachia* DNA from whole flies. For this purpose, we retained the use of the filter and increased the size of one sample from 50 up to 150 specimens in order to obtain a sufficient amount of *Wolbachia*. This combination led to successful enrichment in *Wolbachia* sequences (see Figure S1C,D). The sequence coverage obtained from each data set was enough to be used for genome assembling.

To ensure that our samples do contain *Wolbachia* DNA we have additionally mapped the filtered presumably *Wolbachia* reads onto the wMelCS_b *Wolbachia* reference genome (for details see Section 3.3). The results are shown in Figure 1.While there might be slight differences between our sequenced genomes and the reference genome resulting in underestimation of the *Wolbachia* fraction, we demonstrate that using our method makes it possible to acquire high-quality samples where at least 68.02% of reads for wMelCS^112^ (Figure 1C) and 94.36% of reads for wMelPlus (Figure 1D) can be definitely attributed to *Wolbachia*.

Thus, we demonstrate that our protocol involving only homogenization, differential centrifugation and a single filtration allows us to extract *Wolbachia* DNA from as small a number of *D. melanogaster* imagoes as 150 in an amount and purity sufficient for genomic sequencing.

## 3. Materials and Methods

### 3.1. Drosophila Lines and Rearing

The 11–12 days old females of two *D. melanogaster* lines carrying wMelCS *Wolbachia* strains of different origin, wMelPlus and wMelCS112, on the same nuclear background [17] were used in our study. Flies were maintained on standard medium (agar-agar, 7 g/L; corn grits, 50 g/L; dry yeast, 18 g/L; sugar, 40 g/L) at 25 °C under a 12:12 h light:dark cycle.

### 3.2. DNA Preparation and Sequencing

#### 3.2.1. *Wolbachia* DNA Extraction from *Drosophila* Ovaries

For one sample taken from 50 female fruit flies that were narcotized by CO_2_, ovaries were isolated from the body in a droplet of cold PBS buffer (Invitrogen Corporation, Camarillo, CA, USA) and transferred into a 1.5 mL microtube (Corning Incorporation, Reynosa, Mexico) with 50 μL PBS on ice. Ovaries were homogenized with a sterile pestle and then centrifuged on a Eppendorf Centrifuge 5415R (Hamburg, Germany) for 5 min at 300× *g* and 4 °C to precipitate the *Drosophila*. The supernatant was transferred to a new microtube and centrifuged for 5 min at 4000× *g* and 4 °C to precipitate the *Wolbachia*. The supernatant was removed and directly afterwards DNA extraction was started from the obtained *Wolbachia* pellet using a modified version of the Livak buffer method with ethanol precipitation (80 mM NaCl, 50 mM EDTA, 160 mM sucrose, 130 mM Tris, 0.5% SDS). To achieve that, 100 μL of LIVAK lysing buffer heated up to 65 °C was added to the pellet, then it was resuspended by a pipette and incubated for 30 min at 65 °C in a microthermostate (BIS, M-208, IP Chaldin B.M., Koltsovo, Russia). The mixture was shortly centrifuged to get rid of the condensate droplets, then 14 μL of 8 M potassium-acetate buffer was added and left to chill on ice for 30 min. The obtained mixture was centrifuged and the supernatant was transferred to a new microtube. 200 μL of 99% ethanol was added, the mixture was shaken, then incubated for 2 min at room temperature. The microtube content was centrifuged at 13,000× *g* and 4 °C for 5 min. The supernatant was removed and the pellet was washed twice with 100 μL of 70% iced ethanol with centrifugation for 5 min at 4000× *g* and 4 °C in between. The alcohol was finally removed and the pellet was dried at room temperature. It was dissolved in 20 μL of deionized water and the resulting sample was stored frozen at −20 °C.

#### 3.2.2. *Wolbachia* DNA Extraction from a Whole *Drosophila* Imago

For one sample, 150 female fruit flies were narcotized by CO_2_ and homogenized in a sterile glass homogenizer, with added 20–30 mL of cold buffer containing sucrose, as described by Sun et al. [12] (90 mM KCl, 55 mM CaCl2, 15 mM MgSO4, 30 mM NaCl, 250 mM sucrose). The homogenate was then transferred into a new 50 mL Eppendorf tube and centrifuged in a MIKRO 22 R centrifuge with an angle rotor and adapter 1641 (Hettich, Westphalia, Germany) for 5 min at 80× *g* and 4 °C to precipitate large debris. The supernatant was transferred to a new 50 mL Eppendorf tube and centrifuged for 5 min at 300× *g* and 4 °C to precipitate the remaining debris and *Drosophila* nuclei. The supernatant was once again transferred to a new 50 mL Eppendorf tube and centrifuged at the maximum centrifuge speed of 3824× *g* for 10 min and 4 °C to precipitate bacterial cells. The supernatant was removed, and the pellet was resuspended in 30 μL of cold sucrose-buffer saline. To remove the remaining debris, the homogenate was then centrifuged for 5 min at 300× *g* and 4 °C. The supernatant was passed through a syringe filter with a cellulose acetate membrane and pore diameter of 5 μm (ABLUO, GVS, Bologna, Italy), moistened with a sucrose buffer beforehand using a 50 mL syringe and collecting the supernatant into the new 50 mL Eppendorf tube. The obtained filtrate was centrifuged at 3824× *g* for 15 min and at 4 °C. The supernatant was removed and 0.5 mL of the LIVAK lysing buffer heated up to 65 °C was added to the pellet, then the pellet was resuspended with a pipette, the obtained mixture was transferred to a new 1.5 mL microtube, then incubated for 30 min at 65 °C in the microthermostate. The mixture was shortly centrifuged to get rid of the condensate droplets, then 70 μL of 8 M potassium-acetate buffer was added and left to chill on ice for 30 min. The obtained mixture was centrifuged for 10 min at 15,700× *g* and 4 °C, the supernatant was collected and 1 mL of ethanol was added to the mixture, shaken and then incubated for 2 min at room temperature. The microtube content was centrifuged for 5 min at 15,700× *g* and 4 °C. The supernatant was removed and the pellet was washed twice with 500 μL of 70% iced ethanol. The alcohol was finally removed and the pellet was dried at room temperature. The pellet was then dissolved in 30 μL of deionized water and the resulting sample was stored frozen at −20 °C.

#### 3.2.3. Genome Library Construction and Sequencing

150 ng of DNA was fragmented on a Covaris M220 sonicator 1 n 15 µL tube with parameters optimized for a maximum of fragments sized about 400 bp. Genome libraries were prepared using the Roche KAPA Hyper Prep Kit, KAPA UDI adapters and 50 nanograms of fragmented DNA according to the manufacturer’s manual for dual size selection. Amplification of libraries was carried out in nine cycles. The quality and molarity of the resulting libraries were determined using a Bioanalyzer BA2100. After normalization, barcoded libraries were pooled and sequenced on an Illumina NextSeq550 sequencer (2 × 150 bp) using the NextSeq^®^ 500/550 High Output v2.5 Kit 300 cycles with the expected output for Wolbahia being 30 million reads for the ovarian sample and on an Illumina MiSeq sequencer (2 × 250 bp) using the MiSeq Reagent Kit v2 (Illumina, San Diego, CA, USA) (500-cycles) for the sample from whole flies.

### 3.3. Contamination, Quality Control and Trimming of the Sequenced Data

To evaluate the purity and quality of the DNA samples used for sequencing, the sequence reads were mapped onto the reference genomes (Table 1, please see Table S1 for details) of the *Wolbachia pipientis* wMelCS strain and the probable contaminants such as a host’s nuclear and mitochondrial sequences, human and synthetic sequences (vector contamination, adapters, linkers and primers from the UniVec database). The Illumina reads were filtered using Trimmomatic and mapped onto the genomes of probable contaminants using a Burrows–Wheeler Aligner. The Sequence Alignment Map (SAM)-formatted output file from the Burrows–Wheeler Aligner was converted to the Binary Alignment Map, filtered by mapping quality with SAMtools, and unmapped reads were kept using SeqFilter for subsequent analysis. Standard means for trimming sequenced data and assessing quality were used (FastQC and Trimmomatic).

## Figures and Tables

**Table 1:** Reference genomes against which our sequence data sets were mapped, with their Genbank accession numbers.

RefSeq Assembly Accession	Synonym	Species	Description
GCF_016584405.1	wMelCS_b	*Wolbachia* endosymbiont of *Drosophila melanogaster*	*Wolbachia* reference genome
GCF_000001215.4	dm6	*Drosophila melanogaster*	Release 6 plus ISO1 mitochondrial genome
GCF_000001405.39	hg38	*Homo sapiens*	Genome Reference Consortium Human Build 38 patch release 13 (GRCh38.p13)

## Data Availability

Data have been deposited in the European ENA database; the accession numbers ERR10558042 (NextSeq reads) and ERR10508789 (MiSeq reads) for wMelPlus sample and ERR10558041 (NextSeq reads) and ERR10556501 (MiSeq reads) for wMelCS112 sample.

## Supplementary Materials

The following supporting information can be downloaded at: https://www.mdpi.com/article/10.3390/ijms232315315/s1.
