# Supplementary material for: Easy and Effective Method for Extracting and Purifying Wolbachia Genomic DNA
Source: Int J Mol Sci. 2022 Dec 5;23(23):15315. doi: 10.3390/ijms232315315 (PMC9740973; doi:10.3390/ijms232315315)
Supplement: Supplementary file 1 [file ijms-23-15315-s001.zip › Figure_S1.pptx]

## Slide 1
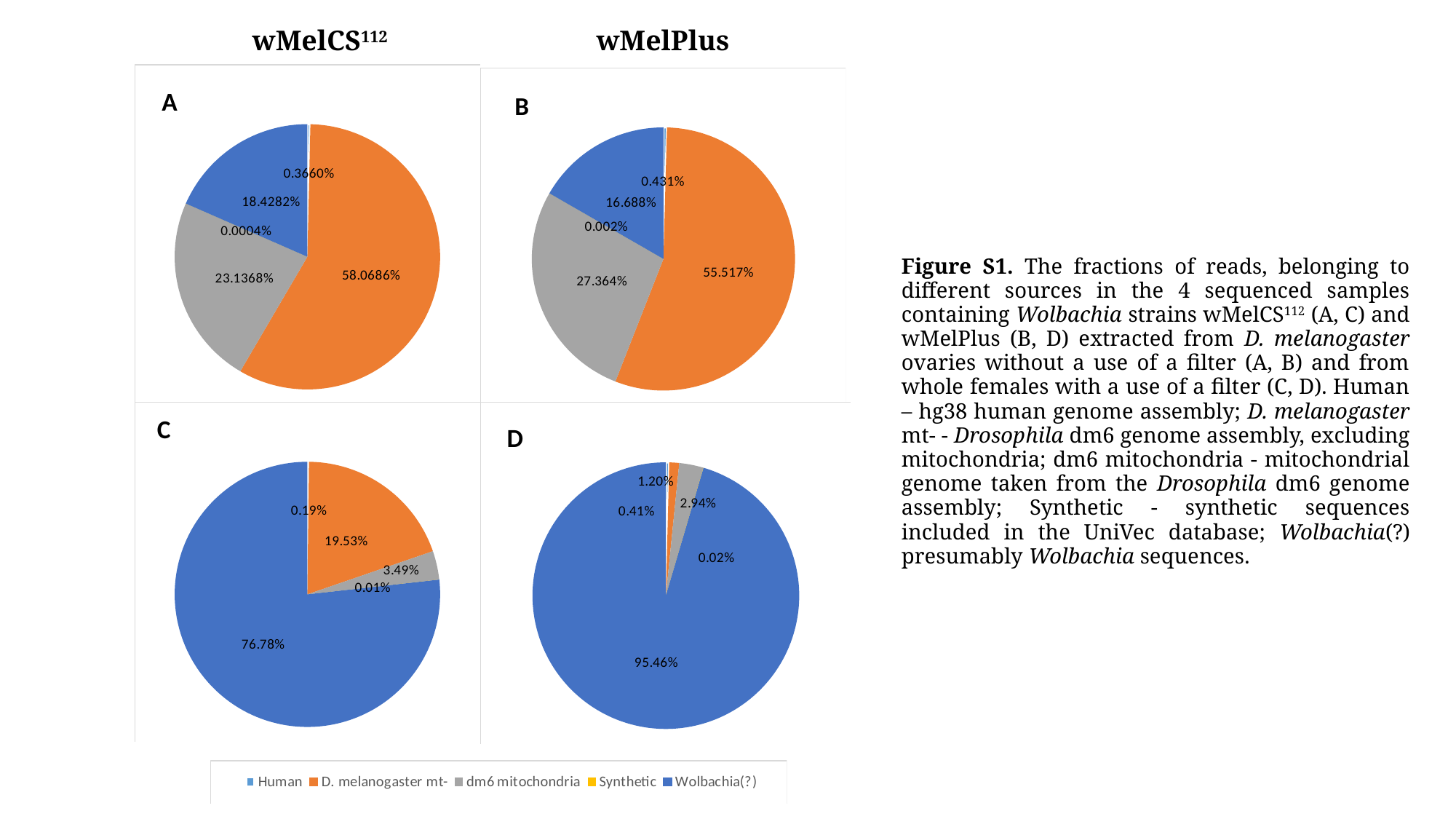

wMelCS112
wMelPlus
Figure S1. The fractions of reads, belonging to different sources in the 4 sequenced samples containing Wolbachia strains wMelCS112 (A, C) and wMelPlus (B, D) extracted from D. melanogaster ovaries without a use of a filter (A, B) and from whole females with a use of a filter (C, D). Human – hg38 human genome assembly; D. melanogaster mt- - Drosophila dm6 genome assembly, excluding mitochondria; dm6 mitochondria - mitochondrial genome taken from the Drosophila dm6 genome assembly; Synthetic - synthetic sequences included in the UniVec database; Wolbachia(?) presumably Wolbachia sequences.
